# The RNA-binding protein RNP29 is an unusual Toc159 transport substrate

**DOI:** 10.3389/fpls.2014.00258

**Published:** 2014-06-16

**Authors:** Julia Grimmer, Anja Rödiger, Wolfgang Hoehenwarter, Stefan Helm, Sacha Baginsky

**Affiliations:** ^1^Plant Biochemistry, Institute of Biochemistry and Biotechnology, Martin-Luther-University Halle-WittenbergHalle (Saale), Germany; ^2^Proteomeanalytik, Leibniz Institute of Plant BiochemistryHalle (Saale), Germany

**Keywords:** plastid protein import, protoplast, RNP29, precursor accumulation, mass spectrometry, import specificity

## Abstract

The precursors of RNP29 and Ferredoxin (Fd2) were previously identified in the cytosol of *ppi2* plant cells with their N-terminal amino acid acetylated. Here, we explore whether precursor accumulation in *ppi2* is characteristic for Toc159 client proteins, by characterizing the import properties of the RNP29 precursor in comparison to Fd2 and other Toc159-dependent or independent substrates. We find specific accumulation of the RNP29 precursor in *ppi2* but not in wild type or *ppi1* protoplasts. With the exception of Lhcb4, precursor accumulation is also detected with all other tested constructs in *ppi2*. However, RNP29 is clearly different from the other proteins because only precursor but almost no mature protein is detectable in protoplast extracts. Co-transformation of RNP29 with Toc159 complements its plastid import, supporting the hypothesis that RNP29 is a Toc159-dependent substrate. Exchange of the second amino acid in the RNP29 transit peptide to Glu or Asn prevents methionine excision but not N-terminal acetylation, suggesting that different N-acetyltransferases may act on chloroplast precursor proteins *in vivo*. All different RNP29 constructs are efficiently imported into wild type but not into *ppi2* plastids, arguing for a minor impact of the N-terminal amino acid on the import process.

## Introduction

Most chloroplast localized proteins are encoded in the nuclear genome and synthesized at cytosolic ribosomes as precursor proteins with N-terminal transit peptides. After import, transit peptides are cleaved by a stromal processing peptidase (SPP) and imported proteins are processed to their mature form (Richter and Lamppa, 1999). Based on training sets with known and established chloroplast proteins, software tools were developed that predict for individual proteins their subcellular localization. Although prediction is error-prone, overall prediction performance for *Arabidopsis thaliana* chloroplast proteins is relatively good, with a true positive prediction rate in the range of 75–85% (van Wijk and Baginsky, 2011). However, sequence features that mediate chloroplast protein import specificity are currently not known (Agne and Kessler, 2009). Recognition and selection of chloroplast-imported proteins are mediated by GTP-binding proteins that belong to two small families: Toc34/33 and Toc159/132/120/90. The Toc159 family members possess a GTP-binding domain (G domain) and a membrane anchoring domain (M domain). They differ by the length of an acidic domain (A domain) that is located N-terminal to the G- and M-domains. Depletion of the major Toc receptors usually results in a defect in photosynthetic growth as demonstrated by decreased accumulation of photosynthetic proteins in *ppi1* and *ppi2* (Jarvis, 2008).

A combination of reverse genetic studies and precursor binding assays suggested two different classes of receptors, one class comprising Toc159/90 and the other class comprising Toc132/120 (Ivanova et al., 2004; Kubis et al., 2004; Jarvis, 2008; Agne and Kessler, 2009; Strittmatter et al., 2010; Schleiff and Becker, 2011). It was proposed that Toc132 and Toc120 are specific for the import of non-photosynthetic proteins while Toc159 and Toc90 are involved in the import of photosynthetic proteins, although this simplified view has been recently challenged (Bischof et al., 2011; Dutta et al., 2014). It is unclear how the different Toc receptors recognize their target proteins but it is conceivable that specificity is mediated by the interaction of Toc-receptor-family members with the transit peptide of precursor proteins (Agne and Kessler, 2009). Functionally relevant amino acid motifs were identified in the RbcS transit peptide but these are not conserved in other photosynthetic proteins (Lee et al., 2008). A recent report suggested that the A-domain of the Toc159 receptor family is involved in mediating precursor selectivity (Inoue et al., 2010). Loss of the A-domain resulted in import receptors with less selective preprotein recognition. This result could explain why over-expression of full length Toc132 or Toc120 failed to complement *ppi2* while constructs containing only the G- and M-domains of Toc132 were able to do so (Inoue et al., 2010).

In an attempt to characterize Toc159 import specificity, Bischof and colleagues performed a comprehensive proteome analysis with Toc159-depleted plant material. The authors identified many photosynthetic proteins that were imported into *ppi2* plastids while many non-photosynthetic functions were affected by the Toc159 mutation, arguing for higher client protein promiscuity than previously anticipated (Bischof et al., 2011). Many proteins whose accumulation was affected in *ppi2* were down-regulated at the transcript level arguing for a complex effect on protein accumulation that does not necessarily indicate the dependence of their import via Toc159. This complex regulation makes it difficult to distinguish true substrates of the Toc159 import pathway from systemic regulation. In fact, a systematic survey for an albino plant-specific proteome phenotype provided evidence that much of the changes in the proteome of albino plants follow common systemic regulation, so that *ppi2* as an albino plant shows typical features of all albino plants including the down-regulation of photosynthetic genes and proteins (Motohashi et al., 2012).

Interestingly, the study by Bischof and colleagues identified precursor proteins that accumulate outside of plastids in the *ppi2* mutant, but not in wild type. This observation was interpreted as a direct consequence of the import defect that would argue for a specificity of Toc159 for the accumulated proteins (Bischof et al., 2011). Usually, precursor proteins are degraded quite rapidly in case they are not imported into plastids, most likely via the ubiquitin proteasome system (UPS) as demonstrated for Lhcb4 (Lee et al., 2009). Notably, Bischof and colleagues found most accumulated precursor proteins N-terminally acetylated. While N-terminal acetylation was assumed to prevent protein degradation since the early 90's it was recently reported as degradation signal for the proteasome in yeast (Hwang et al., 2010). This supports a model in which plastid precursor proteins are modified in the cytosol to decrease their half-life and such avoid their accumulation in an unfolded state. Among these proteins are the known Toc159-dependent protein Ferredoxin and the currently unknown Toc159 target RNP29. RNP29 is a plastid RNA binding protein with two tandem repeat RNA-recognition motifs (RRM) and an N-terminal acidic domain (Lorkovic and Barta, 2002; Kupsch et al., 2012). Using Ferredoxin and RNP29 as a model, we assessed whether their accumulation in *ppi2* is indicative for Toc159 substrate specificity and whether the N-terminal amino acid and its acetylation play a role in protein import.

## Materials and methods

### Plant material

After 2 days of stratification at 4°C *Arabidopsis thaliana* (Columbia-0) and *ppi2* (Toc159, CS11072 introgressed into the Columbia-0 ecotype) (Kubis et al., 2004) were grown on half-strength Murashige and Skoog (M&S) medium supplemented with 0.8% (w/v) plant agar (Duchefa) and 3% (w/v) sucrose under short day conditions at 21°C for 5 weeks before harvesting the seedlings and directly preparing protoplast. Cultivation of *ppi1* (ecotype Wassilewskija) was identical with the exception that M&S medium contained 0.8% (w/v) sucrose. To analyze proteins in wild type and *ppi2* by immunoblotting, plants were grown as described above for 1 week. Afterwards seedlings were transferred into liquid half-strength M&S medium supplemented with 0.8% (w/v) sucrose for further 20 d growth under short day conditions at 21°C.

### Protoplast preparation and transformation

Plants were harvested immediately after the end of the dark period, with root tissue excluded. The plant material was transferred into 400 mM sorbitol, 5 mM MES (pH 5.6), 8 mM CaCl_2_, cut into shreds and incubated after vacuum infiltration in enzyme solution [400 mM sorbitol, 5 mM MES (pH 5.6), 8 mM CaCl_2_, 1.5% (w/v) Cellulase Onozuka R-10 (Serva), 0.375% (w/v) Macerozyme R-10 (Serva)] for 4 h at room temperature in the dark. Protoplasts were released by gentle shaking. After filtration (100 μm BD Falcon™ cell strainer) the number of protoplasts was estimated using a Neubauer chamber. The protoplasts were settled by centrifugation (100×*g*, 5 min) and adjusted to a concentration of 2 × 10^6^ protoplasts per ml in 230 mM NaCl, 187 mM CaCl_2_, 7.5 mM KCl, 7.5 mM glucose, 2.3 mM MES (pH 5.6). After chilling on ice for 30 min the protoplasts were settled again by centrifugation (100×*g*, 5 min) and transferred into 0.4 M sorbitol, 15 mM MgCl_2_, 5 mM MES (pH 5.6) maintaining the concentration of 2 × 10^6^ protoplasts per ml. One hundred microliter of protoplast solution was mixed up with 10 μg plasmid DNA each and 110 μl PEG solution [60% (w/v) PEG4000 (Fluka), 0.3 M sorbitol, 0.15 M Ca(NO_3_)_2_] and incubated for 20 min at room temperature. Protoplasts were washed twice with 230 mM NaCl, 187 mM CaCl_2_, 7.5 mM KCl, 7.5 mM glucose, 2.3 mM MES (pH 5.6) and once with protoplast culture medium (M&S medium, 350 mM sorbitol, 50 mM glucose, 3 mM CaCl_2_, pH 5.8) including 0.1 mg/ml Ampicillin. Transformed protoplasts were stored in protoplast culture medium in darkness.

### Plasmid construction

The vector backbone of all plasmids was pRT100 Ω/Not/Asc (Uberlacker and Werr, 1996) containing the coding sequence of eGFP (Clontech). Wild type Arabidopsis cDNA was used as template to amplify the coding sequence for the first 100 amino acids of the proteins of interest by PCR (Supplementary Figure S1 and Table S1). Sequence was first ligated into pCR2.1®-TOPO® vector (TA cloning®, Invitrogen) and subsequently cloned into the target vector in frame up stream of the eGFP sequence. The plasmids pRT100 Ω/Not/Asc_eGFP as well as pRT100 Ω/Not/Asc_FNR_1−55_:eGFP were provided by Ralf Bernd Klösgen. The plasmid Toc159 inserted in the binary vector pCHF7 that was used for complementation was provided by Birgit Agne and Felix Kessler.

### Fluorescence microscopy

Confocal laser scanning microscopy (CLSM) was performed with a LSM 510 Meta confocal microscope (Carl Zeiss Microscopy, Jena, Germany) at the earliest 20 h after transformation. An argon laser (458, 488, 514 nm) was used, setting an excitation of 488 nm to excite eGFP as well as chlorophyll. Two beam splitters were used: HFT 405/488 and NFT 545, to separate eGFP and chlorophyll fluorescence a BP 505-530 filter and a LP 615 filter were set as well. Images were taken with a Plan-Apochromat 63x/1.40 Oil objective in the channel mode. Pictures were edited using the Zeiss LSM Image Browser.

### SDS-PAGE and western analysis

Protoplast proteins were extracted 23 h after transformation by adding SDS sample buffer [50 mM Tris/HCl (pH 6.8), 2% (w/v) SDS, 10% (v/v) Glycerol, 0.1 M DTT, 0.04‰ Bromphenol blue] and heating the extract for 5 min at 90°C. Every sample represents a doubled transformation reaction. Whole protein extract was separated by SDS-PAGE on 12% polyacrylamide gels and transferred onto polyvinylidene difluoride membranes by semidry blotting. To generate total protein extracts of untransformed wild type and *ppi2*, shock-frozen seedlings were grinded and exposed to Rensink buffer [100 mM NaCl, 50 mM Tris/HCl (pH 7.5), 0.5% (v/v) Triton X-100, 2 mM DTT] including plant protease inhibitor cocktail (Sigma-Aldrich) rotating for 20 min at 4°C. Bradford protein quantification (Bradford, 1976) was done before chloroform/methanol precipitation (Wessel and Flugge, 1984). One hundred fifty microgram protein extract was separated by SDS-PAGE on 12% polyacrylamide gels and transferred by tank blotting onto polyvinylidene difluoride membranes. Immunodetection of proteins was done using enhanced chemiluminescence, and images were obtained by the Fusion Fx7 image-acquisition system (Peqlab). The following antibodies were used: antiGFP (Sigma-Aldrich), antiLhcb4 (Agrisera), antiActin (Sigma-Aldrich) and antiRNP29 [Christian Schmitz-Linneweber (HU Berlin)].

### Sample preparation for MS analyses

SDS-polyacrylamide gels were stained with Coomassie Brilliant Blue. The gel sections corresponding to the apparent molecular weight of proteins of interest were cut. These gel slices were digested with trypsin as previously described (Rodiger et al., 2014). Dried peptides were stored at −20°C for further analyses.

### Nano-LC separation, HD-MS^E^ data acquisition and protein identification/quantification

LC separation and HD-MS^E^ data acquisition was performed as previously described (Helm et al., 2014) using 1 μ l from each of the in gel digested samples, dissolved in 2% (v/v) ACN, 0.1% (v/v) FA, on a ACQUITY UPLC System coupled to a Synapt G2-S mass spectrometer (Waters, Eschborn, Germany). MS acquisition was set to 50–5000 Da. Data analysis was carried out by ProteinLynx Global Server (PLGS 3.0.1, Apex3D algorithm v. 2.128.5.0, 64 bit, Waters, Eschborn, Germany) with automated determination of chromatographic peak width as well as MS TOF resolution. Lock mass value for charge state 2 was defined as 785.8426 Da/e and the lock mass window was set to 0.25 Da. Low/high energy threshold was set to 180/15 counts, respectively. Elution start time was 5 min, intensity threshold was set to 750 counts. Databank search query (PLGS workflow) was carried out as follows: Peptide and fragment tolerances was set to automatic, two fragment ion matches per peptide, five fragment ions for protein identification, and two peptides per protein. Maximum protein mass was set to 250 kDa. Primary digest reagent was trypsin with one missed cleavage allowed. According to the digestion protocol fixed (carbamidomethyl on Cys) as well as variable (acetylation at the N-terminus and oxidation on Met) modifications were set. The false discovery rate (FDR) was set to 4% at the protein level. MS^E^ data were searched against the modified *A. thaliana* database (TAIR10, ftp://ftp.arabidopsis.org) containing common contaminants such as keratin (ftp://ftp.thegpm.org/fasta/cRAP/crap.fasta). Additionally the RNP29_1−100_:eGFP fusion protein as well as the variants A2E and A2N were included. Redundant entries as well as splice variants were removed for database searching. Quantification was performed based on the intensity of the three most abundant proteotypic peptides (Silva et al., 2006). The manual response factor was set to 20,000 counts/fmol.

### Liquid chromatography and mass spectrometry LTQ orbitrap velos pro and peptide/protein identification

Total trypsin protein digest was injected into an EASY-nLCII nano liquid chromatography system (Thermo Fisher Scientific). The peptides were separated using C18 reverse phase chemistry with an EASY-column SC001 pre-column (length 2 cm, inner diameter 100 μm, particle diameter 5 μm) in-line with an EASY-column SC200 (length 10 cm, inner diameter 75 μm, particle diameter 3 μm) both from Thermo Fisher scientific using gradient elution with an organic content increasing linearly from 5 to 40% in 30 min. Peptides were electrospayed on-line into an LTQ-Orbitrap Velos Pro mass spectrometer using a nano-bore stainless steel emitter in a Nanospray Flex ion source all from Thermo Fisher scientific.

The electrospray voltage was set to 1.9 kV, the capillary temperature to 275°C, the RF Lens level to 50% and the difference in multipole offset to −7 V to ensure a stable electrospray with a current around 1 μA. Both ion trap (IT) and Orbitrap (FT) injection waveforms were enabled. FT mass spectra were internally calibrated on the fly with the lock mass function using the ambient mass 445.1200. A data dependent acquisition (DDA) method with an inclusion list was used to isolate, fragment and record MS/MS spectra of only ions on the inclusion list with the 20 most intense signals in a scan of the total ion population (MS full scan) in the Orbitrap mass analyzer using collision induced dissociation (CID) in the linear trap quadrupole (LTQ) mass analyzer. The precursor mass tolerance was ±10 ppm. One microscan was acquired for both MS full and MS/MS scans. The minimum precursor ion signal intensity threshold was set to 1000, the isolation width to 2 Da. The automatic gain control (AGC) was set to 1e+06 for the Orbitrap and 1e+04 for the LTQ mass analyzer; the maximum injection times were 500 and 200 ms, respectively.

Alternatively, a targeted method employing an MS full scan followed by MS/MS acquisition of all ions on the global mass list irrespective of their signal intensity in the preceding full scan in the LTQ was used. The isolation width was set to 3 Da, the other parameters were as above.

Raw files from the mass spectrometer were imported into the Proteome Discoverer v.1.4 mass analysis environment (PD) from Thermo Fisher Scientific. Database search of the TAIR10 database with target proteins and common contaminants added (32,793 sequences, 14,486,974 residues) was conducted using the Mascot software v2.4.0 connected to PD to identify peptides and proteins. For peak list generation, a signal to noise threshold of 1.5 was used to filter peaks from MS full scans. For the database search, the precursor ion tolerance was set to 7 ppm, the fragment ion tolerance to 0.8 Da. The enzyme was set to trypsin, 2 missed cleavages were tolerated. Protein N-terminal acetylation was set as a variable modification, phosphorylation of serine and oxidation of methionine were included in alternative searches; carbamidomethylation of cystein was set as a fixed modification. The family wise PSM error rate was controlled with FDR/q-values using the reversed target/decoy database model of the null hypothesis for PSM with the target decoy PSM validator module in PD.

## Results

### Acetylation of plastid precursor proteins in the cytosol of the *ppi2* mutant

We have previously reported N-terminal acetylation of precursor proteins in the cytosol of the plastid protein import deficient mutant *ppi2* (Bischof et al., 2011). To analyze the sequence context of N-terminal precursor acetylation, we extracted from the previous dataset all identified acetylated precursor proteins (Table 1). In the list of 13 acetylated precursors, all carry an alanine in the second amino acid position following the initiator methionine. Furthermore, nine of the 13 precursors carry another alanine in the third position (Table 1), while the remaining four carry serine, leucine, valine, or glutamate in position three. We identified the N-terminal peptide exclusively with the initiator methionine removed and in all cases only the acetylated, but not the non-acetylated precursor was detected (Bischof et al., 2011). This observation is consistent with a co-translational acetylation process that operates on nascent precursor proteins that fulfill the sequence requirements for acetylation. In yeast, the alanine in position two typically triggers methionine excision (Sherman et al., 1985) that is a prerequisite for N-terminal acetylation by A-type N-acetyltransferases (NatA) (Polevoda and Sherman, 2003; Martinez et al., 2008). In analogy to the yeast system, our data suggest that a NatA-type enzyme may be responsible for precursor acetylation (Hollebeke et al., 2012). Using a chloroplast proteome reference table (Reiland et al., 2009; van Wijk and Baginsky, 2011) we analyzed chloroplast proteins for the occurrence of alanine at position two and three in the transit peptide. Out of 1524 nucleus-encoded chloroplast precursor proteins, 746 carry an alanine in position two (49%), of which 131 (17.5%) carry an alanine also in position three. Our dataset reported above is thus highly enriched (69.2%) for MAA- containing transit peptides suggesting high substrate specificity for the acetylation reaction (Table 1).

**Table 1:** **N-terminally acetylated peptides in chloroplast precursor proteins**.

**Identifier**	**Description**	**Acetylated peptide**	**Acetylation position**	**Identified in mutant**	**Spectral count (acetyl/non-acetyl)**
AT1G10960	Ferredoxin	ASTALSSAIVSTSFLR	2	*ppi2*	2/0
AT2G37220	29 kDa ribonucleoprotein	AASASSLALSSFNPK	2	*ppi2*	5/0
AT3G12780	Phosphoglycerate kinase	ASAAASSAFSLLK	2	*ppi2*	7/0
AT4G25100	Fe-superoxide dismutase	AASSAVTANYVLKPPPFALDALEPH	2	*ppi2*	25/0
AT5G54770	THI1	AAIASTLSLSSTKPQR	2	*ppi2*	27/0
AT5G66570	PSBO1	AASLQSTATFLQSAK	2	*ppi2*	2/0
AT2G39730	Rubisco activase	AAAVSTVGAINR	2	*ppi2*	6/0
AT3G23920	Beta-amylase	ALNLSHQLGVLAGTPIK	2	*ppi2*	2/0
AT5G20720	CPN20	AATQLTASPVTMSAR	2	*ppi2*	1/0
AT5G66040	Sulfurtransferase protein 16	AEESRVPSSVSVTVAHDLLLAGHR	2	*ppi2*	1/0
AT3G54050	Fructose-1,6-bisphosphatase	AATAATTTSSHLLLSSSR	2	Toc159cs	2/0
AT5G53850	Haloacid dehalogenase-like	AVAAAAMIGLPQAYLEGK	2	Toc159cs	2/0
AT1G61520	LHCA3	AAQALVSSSLTSSVQTAR	2	Toc159cs	1/0

*The list was generated from data obtained by Bischof et al. (2011). All putative plastid proteins with conflicting reports on subcellular localization (e.g., as reported in SUBA, http://suba.plantenergy.uwa.edu.au/, including those proteins that are dual targeted were removed from the list)*.

### RNP29—an unusual substrate of Toc159

MAA- containing precursor proteins may accumulate in the cytosol of *ppi2* plants, because they are direct substrates for Toc159 and cannot be imported in its absence. In order to address this question, we selected identified precursors and characterized their import properties in a protoplast system in greater detail. We selected RNP29, because (i) it starts with MAA- and as such represents a typical acetylation substrate and (ii) it represents a new Toc159 substrate because as RNA-binding protein it does not conform to the previous assumption that Toc159 is mainly involved in the import of photosynthetic proteins (Bauer et al., 2000). For characterization of RNP29 import properties, we fused the 100 most N-terminal amino acids including the transit peptide with an eGFP reporter protein and transformed wild type and *ppi2* protoplasts with these constructs. Using a combination of microscopy and western blotting, we established plastid protein import by co-localization of eGFP and chlorophyll fluorescence and by the mobility shift that is induced upon protein import by proteolytic processing. Since the specific cleavage of the transit peptide occurs exclusively in the plastid, a difference in size between preprotein and mature protein strongly suggests correct protein import (Zybailov et al., 2008; Bischof et al., 2011).

**Figure 1:**
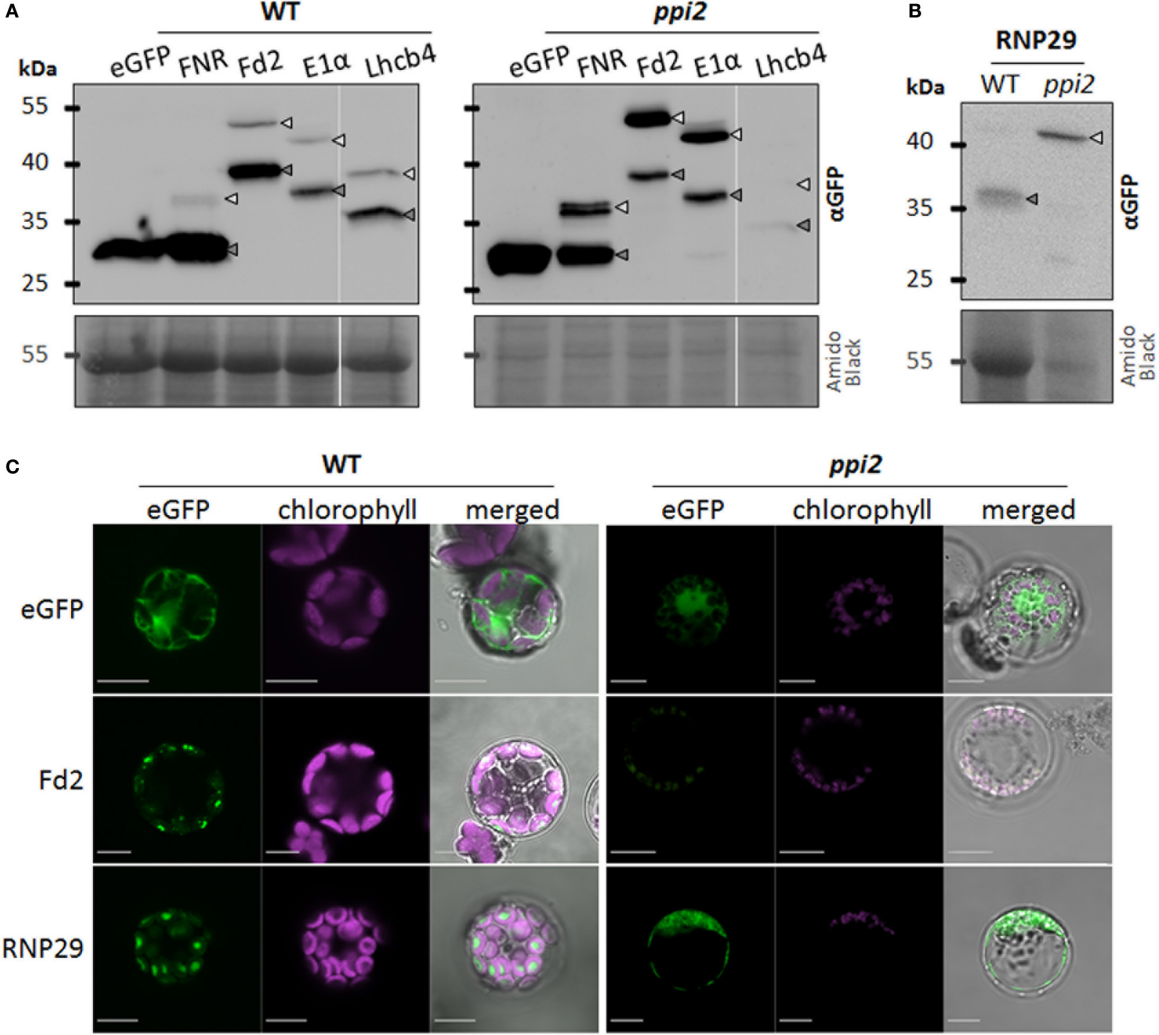
***In vivo* import of eGFP fusion proteins in the TOC159 deficient mutant *ppi2* and in wild type. (A)** And **(B)** Western blot analysis of wild type (WT) and *ppi2* protoplasts transiently expressing eGFP or the indicated eGFP fusion protein using an anti-GFP antibody; arrows indicate positions of preproteins (light gray) and mature proteins (dark gray). The amido black stained membrane is shown as loading control. **(C)** CLSM of WT and *ppi2* protoplasts transiently transformed with eGFP, Fd2_1-100_:eGFP or RNP29_1-100_:eGFP, bars = 10 μm (further information in Supplementary Figure S2).

The protoplast protein import data are presented in Figure 1. We compared the RNP29 import characteristics with those of Fd-dependent NADP reductase (FNR), Ferredoxin, pyruvate dehydrogenase E1α and Lhcb4 eGFP fusion proteins. Ferredoxin was chosen as substrate because it was also found as acetylated precursor protein in *ppi2* (Table 1) and its Toc159-dependent import was previously reported (Smith et al., 2004). Since it carries a serine in position three, it does not conform to the MAA- N-terminus of acetylated precursor proteins (Table 1). The E1α transit peptide carries a threonine in position two and its import is thought to be Toc159-independent (Bischof et al., 2011). In contrast, Lhcb4 carries an MAA-N-terminus and its import was found to be Toc159-dependent (Lee et al., 2009). However, Lhcb4 was not identified as an acetylated precursor in *ppi2* by Bischof and colleagues, suggesting further diversification among putative Toc159 client proteins (Bischof et al., 2011).

Transiently expressed eGFP without any transit peptide is localized in the protoplast cytosol, whereas eGFP fused to the transit peptide of spinach FNR is efficiently transported into and processed in chloroplasts of wild type (Figure 1A). Cleavage of the transit peptide shifts the apparent size of the fusion protein FNR_1−55_:eGFP from 36 to 27 kDa (Figure 1A). A fusion of the first 100 amino acids of plastid localized proteins Fd2, E1α, and Lhcb4 with the eGFP reporter also results in a chloroplast localization of the fusion proteins and a shift of protein size in the western blot analysis. Thus, all precursor constructs are efficiently imported into wild type chloroplasts (Figure 1A).

Surprisingly, a similar picture was obtained with *ppi2* protoplasts with the exception that precursor proteins accumulate to a much higher abundance compared to the mature protein (Figures 1A,B). This entails FNR_1−55_:eGFP, Fd2_1−100_:eGFP and E1α_1−100_:eGFP. While the plastid localization of the eGFP constructs in *ppi2* protoplasts is hard to prove by microscopy (Figure 1C), the western blot analysis clearly shows successful import of all fusion proteins into plastids of *ppi2* (Figure 1A). In comparison to wild type, the ratio of unprocessed and processed FNR_1−55_:eGFP, Fd2_1−100_:eGFP and E1α_1−100_:eGFP is significantly shifted toward the preprotein. In *ppi2* protoplasts a small amount of mature Lhcb4 was detected by western blot analysis, while Lhcb4 preprotein was not identified. This can be explained either by efficient degradation of Lhcb4 precursor by the UPS as a result of inefficient import or by a low transformation rate particularly with this construct.

**Figure 2:**
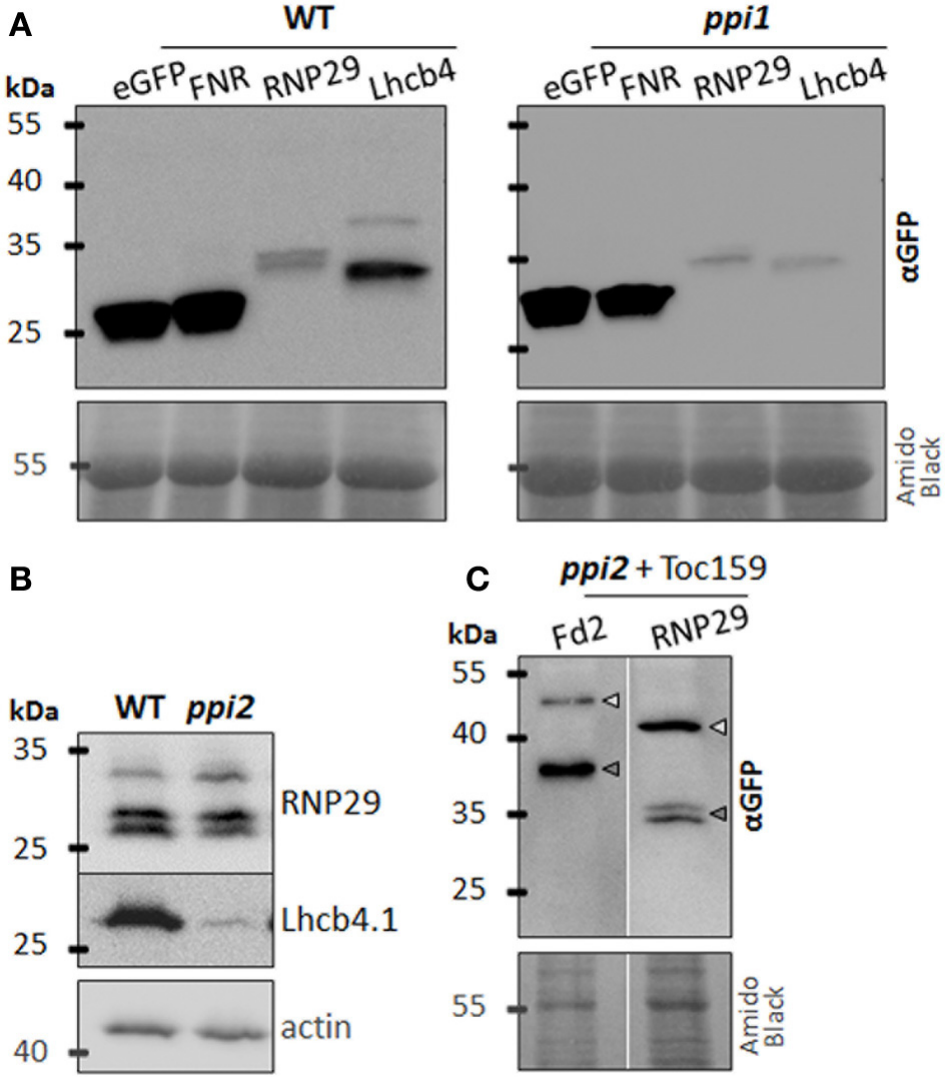
**RNP29 import is Toc 159 dependent. (A)** Western blot analysis of wild type (WT) and *ppi1* protoplasts transiently expressing eGFP or the indicated eGFP fusion protein containing at least the transit peptide of plastid localized FNR, RNP29, or Lhcb4. Detection was achieved by an anti-GFP antibody. The amido black stained membrane is shown as loading control. **(B)** Abundance of cpRNPs 29A (At3g53460, upper band) and 29B (At2g37220, lower double band) and Lhcb4.1 (At5g01530) in total protein extracts from wild type (WT) and *ppi2* seedlings. In total 150 μg protein was loaded and actin was used as loading control. **(C)** Western blot analysis of reconstituted import of eGFP fusion proteins Fd2_1-100_:eGFP and RNP29_1-100_:eGFP in *ppi2* protoplasts cotransformed with full length Toc159. The reporter protein was detected with an anti-GFP antibody; arrows indicate the positions of preproteins (light gray) and mature proteins (dark gray). The amido black stained membrane is shown as loading control.

The data obtained with the RNP29 construct support its Toc159-dependent import into plastids. While the construct is efficiently imported into wild type chloroplasts, only the precursor but not the mature form of RNP29 is detectable in *ppi2* protoplasts (Figure 1B). None of the control constructs shown in Figure 1A, revealed such a distinctive difference between wild type and *ppi2* import properties. The RNP29 precursor appears quite stable in *ppi2* protoplasts, similar to the FNR-, Fd2- and the E1α-precursor that are readily detectable in *ppi2* protoplasts but in contrast to the Lhcb4 precursor (Figures 1A,B). The FNR_1−55_:eGFP, Lhcb4_1−100_:eGFP and RNP29_1−100_:eGFP constructs were also transiently expressed in *ppi1* mutant protoplasts, that are devoid of Toc33. The Lhcb4_1−100_:eGFP construct shows a similar accumulation pattern as in the *ppi2* mutant most likely for the same reasons as discussed above. In contrast to the *ppi2* mutant, the FNR_1−55_:eGFP and the RNP29_1−100_:eGFP constructs are efficiently imported into *ppi1* plastids and precursor proteins are not detectable (Figure 2A). This supports the conclusion that the defect in RNP29 import is specifically due to the lack of Toc159. Again, the low amount of detectable mature protein in *ppi1* could result from degradation of non-imported protein or low transformation rates. Nonetheless the fact that only mature protein but not precursor protein can be detected suggests that Toc159 can form import competent Toc complexes that are independent of Toc33, which explains the weaker phenotype of *ppi1* compared to *ppi2* (Kubis et al., 2004).

We analyzed the accumulation of RNP29 and Lhcb4 in wild type and in *ppi2* seedlings by western blotting. Consistent with our protoplast assays, Lhcb4 accumulates to much lower levels in *ppi2* compared to wild type and only the mature form but no precursor was found to accumulate (Figure 2B). The low Lhcb4 accumulation in seedlings may partially result from transcriptional regulation that we can exclude for the Lhcb4 construct in *ppi2* protoplasts, because we used the 35S promoter to trigger its expression. The low accumulation of mature Lhcb4 is therefore probably also partly due to posttranslational degradation as a consequence of inefficient import, e.g., by the UPS as suggested earlier (Lee et al., 2009). Surprisingly, mature RNP29 accumulates to similar levels in wild type and in *ppi2* plastids (Figure 2B). We cannot identify the precursor of RNP29 in this western blot because its molecular mass is almost identical to the molecular mass of RNP29A that is also recognized by this antibody (Figure 2B) (Schmitz-Linneweber, personal communication). A possible explanation for the accumulation of a mature Toc159-dependent substrate in *ppi2* plastids could be a developmental re-organization of the import machinery that allows RNP29 to enter the plastid during early stages of development via a Toc159-independent route (Teng et al., 2012) (see Discussion).

The result of the RNP29 antibody blot creates a paradox that requires further attention. It is unlikely that the accumulation of RNP29 precursor as reported above is a protoplast artifact, because this protein is readily imported into wild type and *ppi1* plastids and because precursor accumulation was originally found in *ppi2* seedlings, suggesting that this precursor also accumulates under *in vivo* conditions (Bischof et al., 2011). In order to lend further support to or reject the hypothesis that Toc159 is responsible for RNP29 import, we co-transformed the RNP29_1−100_:eGFP with a Toc159 construct into *ppi2* protoplasts. In the co-transformed protoplasts, import of RNP29 is complemented and mature RNP29 accumulates (Figure 2C), albeit to a much lower extend compared to wild type (Figure 1B). Thus we conclude that Toc159 is required for the import of the RNP29 preprotein. This support the hypothesis that precursor selectivity of the Toc receptors depends on physico-chemical properties of the protein and/or the transit peptide that are not restricted to photosynthetic proteins (Bischof et al., 2011; Dutta et al., 2014). It is not clear which sequence properties mediate the Toc159-dependent import of RNP29 but as the list of Toc159 client proteins grows, a statistical assessment to pinpoint such characteristics will become feasible.

### The N-terminal amino acid does not affect protein import efficiency or precursor accumulation

**Figure 3:**
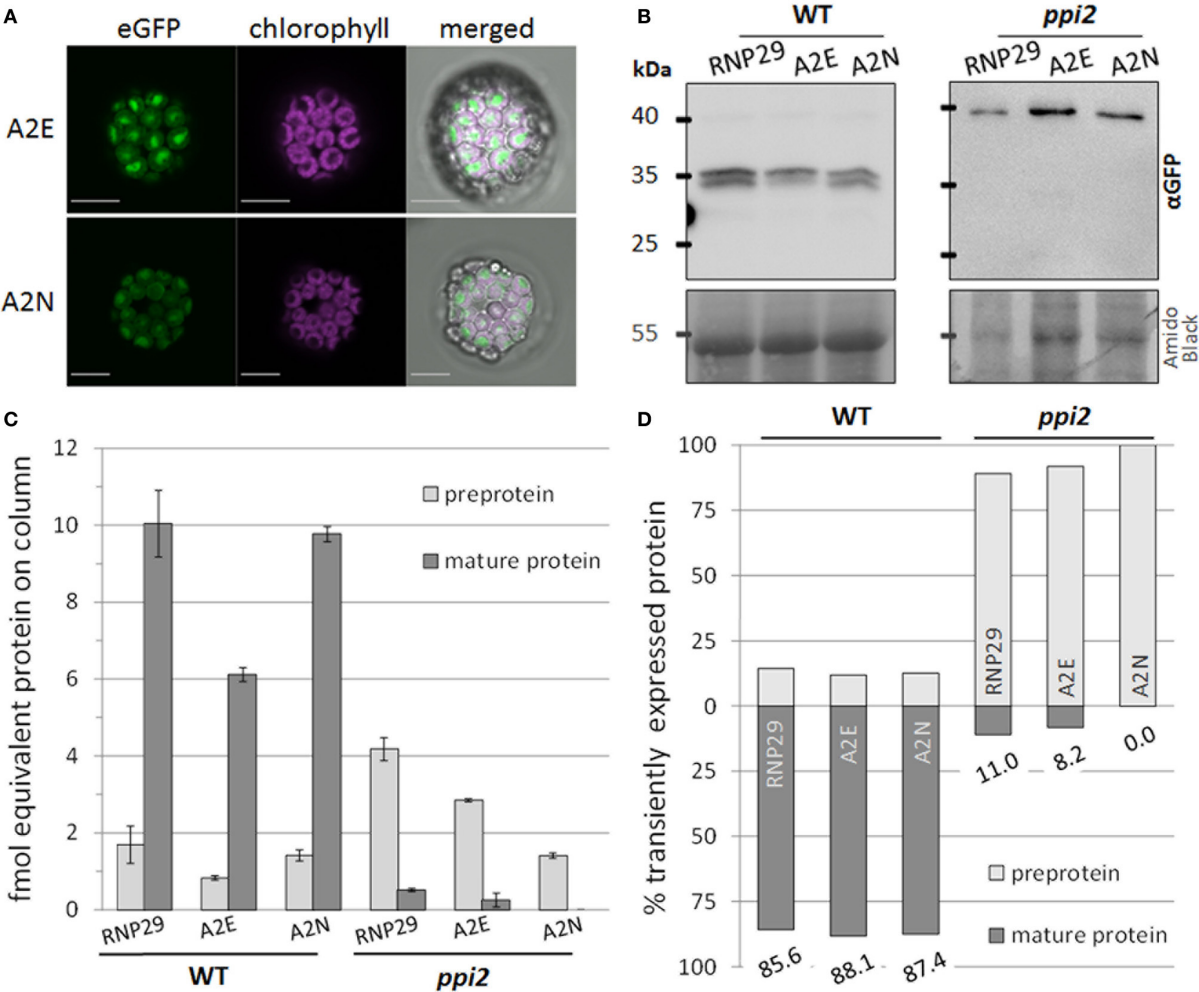
**Import efficiency of different RNP29 constructs**. The second amino acid of RNP29_1-100_:eGFP (RNP29) Ala was replaced by Glu in A2E or Asn in A2N. Import efficiency of the substrates was analyzed in transiently transformed wild type (WT) and *ppi2* protoplasts with different methods, showing the import efficiency of the A2E and the A2N construct. **(A)** CLSM of transiently transformed protoplasts, scale bars = 10 μm. **(B)** Western blot analysis using an anti-GFP antibody. The amido black stained membrane is shown as loading control. **(C)** and **(D)** Quantification of preprotein and mature protein using MS^E^. Diagrams showing the absolute quantification with standard deviation **(C)** and the relation between preprotein and mature protein **(D)** for each construct in wild type (WT) and *ppi2*.

To reveal the effect of the N-terminal amino acid and its acetylation on RNP29 import, we compared the import efficiency between different RNP29 precursor constructs. Since precursor acetylation shows all characteristics of the combined action of methionine aminopeptidase and NatA-type enzymes, we reasoned that an exchange of the second amino acid should sufficiently alter the properties of the nascent precursor to decrease the efficiency of methionine cleavage and N-acetylation by A-type N-acetyltransferases. To this end, we exchanged Ala in position two (in RNP29_1−100_:eGFP) to Glu (RNP29_1−100_A2E:eGFP) or Asn (in RNP29_1−100_A2N:eGFP). In wild type protoplasts, mature RNP29 was identified with all three constructs, while in *ppi2* protoplasts only the precursor proteins are detectable (Figures 3A,B). This accumulation characteristic is consistent between the three constructs and no obvious alterations in response to the amino acid exchange at position two are visible. The eGFP reporter is clearly located in the chloroplast of wild type protoplasts with no obvious difference between RNP29_1−100_:eGFP, RNP29_1−100_A2E:eGFP and RNP29_1−100_A2N:eGFP (Figure 3A). The accumulation of the three constructs differed in different biological replicates, but the differences were not consistent between the replicates.

It is likely, that differences in the abundance of RNP29 result from differences in the protoplast transformation and protein expression rates. Thus, a comparison of import efficiency between the different constructs requires a relative measure independent of the transformation rate. We therefore determined the ratio between mature RNP29 and precursor protein by quantitative mass spectrometry via HD-MSE (Helm et al., 2014). The measured amount of total transiently expressed protein varies between 11.7 and 6.9 fmol in wild type and 4.6 and 1.4 fmol in *ppi2* protoplasts (Figure 3C). The ratio between preprotein and mature RNP29 differs dramatically between wild type and *ppi2*, but no consistent differences between the constructs within one genotype were observed. For the presented wild type replicate, we determined a stable amount between 85 and 88% of total RNP29 to be correctly imported while only between 0 and 11% mature protein were identified in *ppi2* (Figure 3D). The MS-based quantification is consistent with the western blot analyses and allows us to conclude that RNP29 import efficiency is not affected by the single amino acid exchange at position two (Figures 3B–D).

For the interpretation of the results it is important to know whether the amino acid exchange prevented methionine cleavage and/or N-terminal acetylation as expected (see above). To address this question, we digested the gel band containing the precursor protein with trypsin and searched for the N-terminal peptide by inclusion mass scanning on an Orbitrap-Velos. We allowed different combinations of methionine removal, with or without methionine oxidized, and with or without acetylation. Surprisingly, we identified the three N-terminal precursor peptides in their acetylated form (Table 2 and Supplemental Figure S3). The wild type construct had its methionine removed and its alanine acetylated, as expected. Also here, no non-acetylated peptides were identified. The A2E and A2N constructs retained their N-terminal methionines, and were still found to be acetylated. Thus, although we managed to prevent methionine cleavage with the amino acid exchange, we did not prevent N-terminal acetylation suggesting that either different types of N-acetyltransferases act on plastid precursor proteins or that the Arabidopsis enzymes have broader substrate specificity compared to the yeast system.

**Table 2:** **N-terminal modifications of RNP29 constructs identified in *ppi2* protoplasts**.

**Protein**	**N-terminal peptide**	**Modifications**	**# PSMs**	**Ionscore**
RNP29	aASASSLALSSFNPK	N-Term(Acetyl)	36	94.11
A2E	mEASASSLALSSFNPK	N-Term(Acetyl)	15	74.46
A2N	mNASASSLALSSFNPK	N-Term(Acetyl)	2	52.12

*A targeted MS approach resulted in the identification of acetylation and methionine excision of the authentic RNP29 N-terminal peptide. The modified peptides A2E and A2N were acetylated at the start methionine. The peptide spectral matches (PSMs) and the ion score were generated by the Mascot software*.

## Discussion

Here we establish RNP29 as Toc159-dependent client protein, which is in line with the original interpretation of RNP29 precursor accumulation in *ppi2* plastids (Bischof et al., 2011). RNP29 reveals characteristics that are similar to the already known Toc159 client protein Fd2 (Table 1) because the import of both proteins is facilitated by co-transformation with Toc159 (Figure 3C). In contrast to Ferredoxin, the accumulation of RNP29 as a precursor is much more pronounced and almost no mature protein is detectable in *ppi2* protoplasts, while imported Ferredoxin is readily detectable (Figures 1A,B). This is surprising in light of the western blot results that showed endogenous RNP29 protein amounts in wild type and in *ppi2* plants up to comparable levels (Figure 2B). The specificity of the import machinery changes during development and it is conceivable that its re-organization adjusts import efficiency and specificity to prevailing requirements (Li and Teng, 2013). It is possible, that RNP29 enters the plastid via Toc159-independent routes in *ppi2* that are in place at a certain developmental stage. This possibility is supported by the observation that RNP29 expression peaks at around 96 h after germination, suggesting that the mass import of RNP29 occurs early in development (Wang et al., 2006). It is furthermore possible, that low efficiency import of RNP29 occurs throughout plastid development via other Toc receptors. Provided that sufficient time is available for import, proteins such as RNP29 may enter the plastid in an unspecific manner and accumulate to near wild type amounts. Such a scenario is supported by the observation that around 11% of total expressed RNP29 fusion protein is imported into *ppi2* plastids (Figure 3D).

A stable cytosolic precursor as well as a high expression level support a long residence time of RNP29 in the cytosol. We know that RNP29 is not down-regulated at the transcriptional level in *ppi2*, and the fact that we identified it as one of very few precursors in the cytosol argues for its relatively high stability (Bischof et al., 2011). In the artificial protoplast system, we find most precursor proteins to accumulate in *ppi2*, with the exception of Lhcb4 (Figure 1). Thus, these precursors are relatively stable in the cytosol and their lack of detection in *ppi2* seedlings is probably a result of fine-tuned transcriptional regulation (Bischof et al., 2011). This is clearly different for Lhcb4. While most photosynthetic proteins are down-regulated at the transcriptional level in *ppi2* and other albino plants, we can exclude transcriptional regulation for Lhcb4 in our artificial protoplast system. The lack of Lhcb4 precursor detection here (Figure 1) therefore argues for a tight quality control system that affects certain types of precursor proteins, degrading them efficiently before they can accumulate. Since such degradation occurs for Lhcb4, but not for other precursors in our protoplast system, we conclude that different degradation- or stabilization-mechanisms exist for precursor proteins in the cytosol.

N-acetylation is a common co-translational modification in higher eukaryotes that is mediated by N-acetyltransferases (NATs). The second amino acid is crucial for substrate specificity of NATs in yeast and mammalian systems, and it is conceivable that a similar mechanism exists in plants (Hollebeke et al., 2012). More than 50% of the plastid precursor proteins carry an alanine in position two, making them typical NatA substrates. NatA activity depends on N-terminal methionine excision suggesting that an exchange of Ala to Glu or Asn at position two would not only abolish methionine cleavage but also N-terminal acetylation by an A-type NAT. For RNP29 we find that the exchange of the second amino acid indeed eradicates methionine cleavage but not N-terminal acetylation (Table 2). This is surprising and suggests that not only NatA-type enzymes act on plastid precursor proteins, but also NatB-type enzymes. Only very little is known about the substrate specificity and the function of N-acetyltransferases in plant systems. Recently, a loss of function mutant in a non-catalytic component of a NatB-type enzyme complex was characterized. The analysis revealed pleiotropic phenotypes including changes in flowering time regulation and leaf, inflorescence, flower, fruit and embryonic development (Ferrandez-Ayela et al., 2013). So far, the only cytosolic N-acetyltransferase with a known effect on chloroplast development is AtMak3 that resembles NatC-type enzymes. Its defect results in delayed chloroplast development, however, its target protein spectrum and its functions are currently unknown (Pesaresi et al., 2003).

At present, the role of precursor acetylation in the import process remains elusive. Similar to the yeast system, precursor acetylation may be a *degron* that ensures low residence time of non-imported plastid proteins in the cytosol (Hwang et al., 2010). While this would be an elegant possibility, there is currently no indication that N-terminal acetylation serves as *degron* in plants. In contrast, acetylation of proteins in the chloroplast stroma in Chlamydomonas even increases their half-lives (Bienvenut et al., 2011). Furthermore, N-terminal acetylation could affect the interaction of the N-terminal transit peptide region with heat shock proteins. One of the few identified functional regions in transit peptides is a short, uncharged N-terminal segment that seems capable to function as Hsp70-binding domain. This interaction is important for the formation of translocation intermediates, thus any change in the interaction properties could affect the import of precursor proteins (Chotewutmontri et al., 2012). Acetylation in this region could strengthen the interaction of precursor with heat shock proteins and thus either determine the efficiency of precursor interaction and/or even the specificity of import. We are currently investigating these different possibilities.

Recent attempts to identify functionally relevant domains in transit peptides were not successful, even after grouping proteins into relevant categories such as “Toc159-dependent” and “Toc159-independent” (Bischof et al., 2011). This is probably because transit peptides contain different modules that can be arranged in a diverse order (Li and Teng, 2013). Thus, any assembly of proteins at larger-scale will most likely average out an otherwise significant enrichment of amino acids in a functional domain. Comparing the transit peptides of Toc159-dependent client proteins such as Fd2 and RNP29 with the Toc159-independent E1α and FNR transit peptide, we find for the latter a smaller uncharged N-terminal region that is interrupted by lysine, an occurrence of unusual amino acids such as glutamic acid in E1α and aspartic acids in FNR as well as histidine proximal to the N-terminus, followed by a more pronounced stretch of hydrophobic amino acids in the E1α transit peptide sequence. In the RNP29 transit peptide, a putative degenerated FGLK motif is found in two positions, one around amino acid 36 that is consistent with its position in other transit peptides (Chotewutmontri et al., 2012). This one is lacking glycine as the “helix-breaking” amino acid in its closer surrounding. A complete motif is found at position 20. Whether or not this has any relevance for the observations we made here remains unclear. Further experiments are necessary to understand the design of transit peptides. Sufficient hypotheses are available and await further testing (Li and Teng, 2013).

### Conflict of interest statement

The authors declare that the research was conducted in the absence of any commercial or financial relationships that could be construed as a potential conflict of interest.
