# Supplementary material for: The RNA-binding protein RNP29 is an unusual Toc159 transport substrate
Source: Front Plant Sci. 2014 Jun 16;5:258. doi: 10.3389/fpls.2014.00258 (PMC4059279; doi:10.3389/fpls.2014.00258)
Supplement: Supplementary file 1 [file DataSheet1.DOCX]

**Supplemental Material**


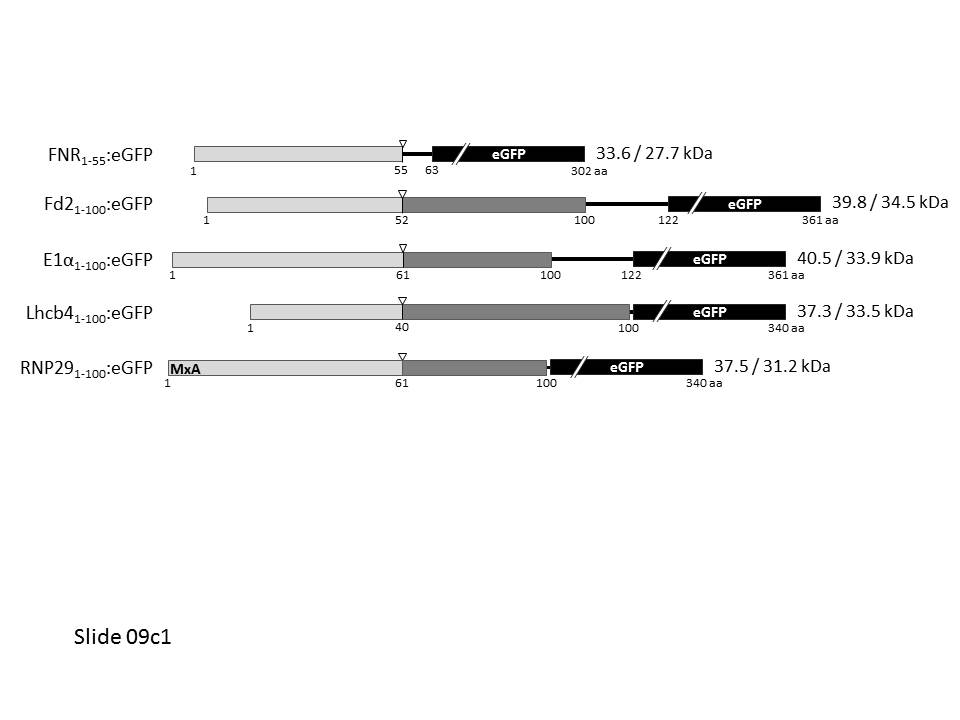


**Supplemental Figure S1: Scheme of the fusion proteins used for protoplast transformation**

Transiently expressed proteins contain at least a transit peptide sequence (light grey), a 1 to 22 amino acid linker (black bar) and the eGFP reporter. The predicted cleavage site of the transit peptide is marked with an arrow (predicted by UniProtKB ([www.uniprot.org](http://www.uniprot.org))), calculated molecular weights are provided (unprocessed/ processed, calculated by the ExPASy tool ([www.expasy.org)](http://www.expasy.org))). FNR_1-55_:eGFP includes 55 amino acids of the spinach FNR transit peptide. All other proteins consist of the first 100 amino acids of the *A. thaliana* amino acid sequence. The second amino acid of RNP29_1-100_:eGFP is varied containing Ala (native), Glu or Asn.


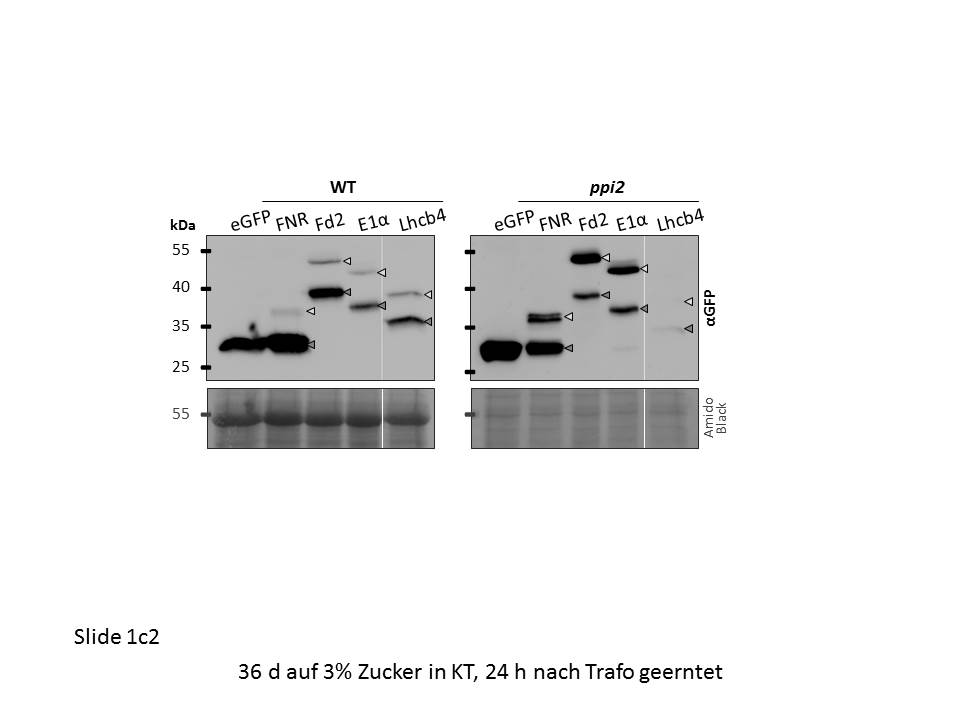

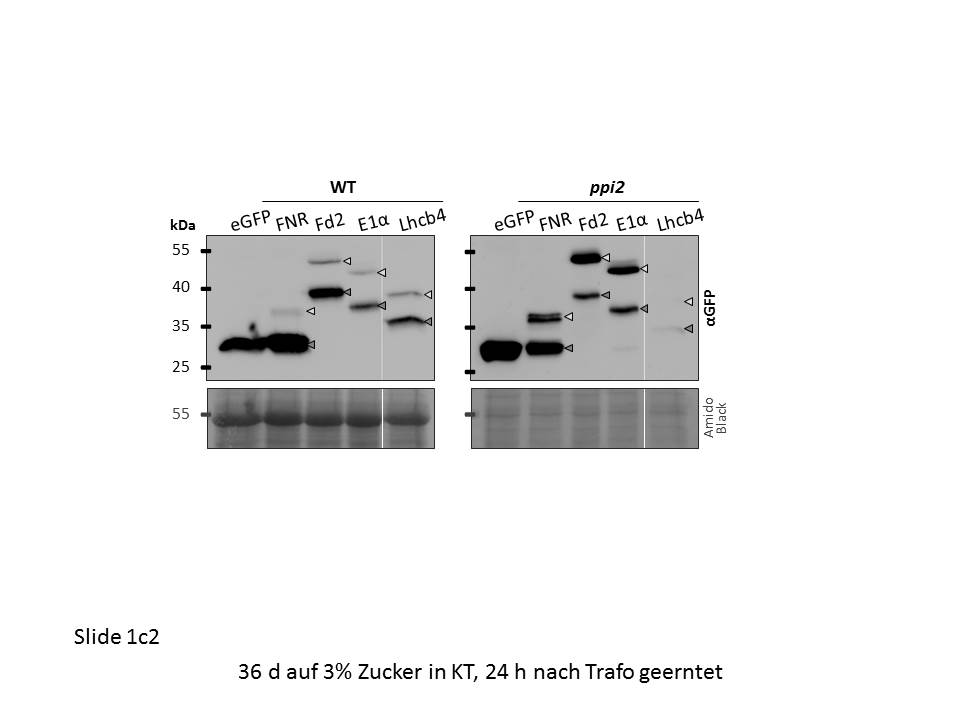

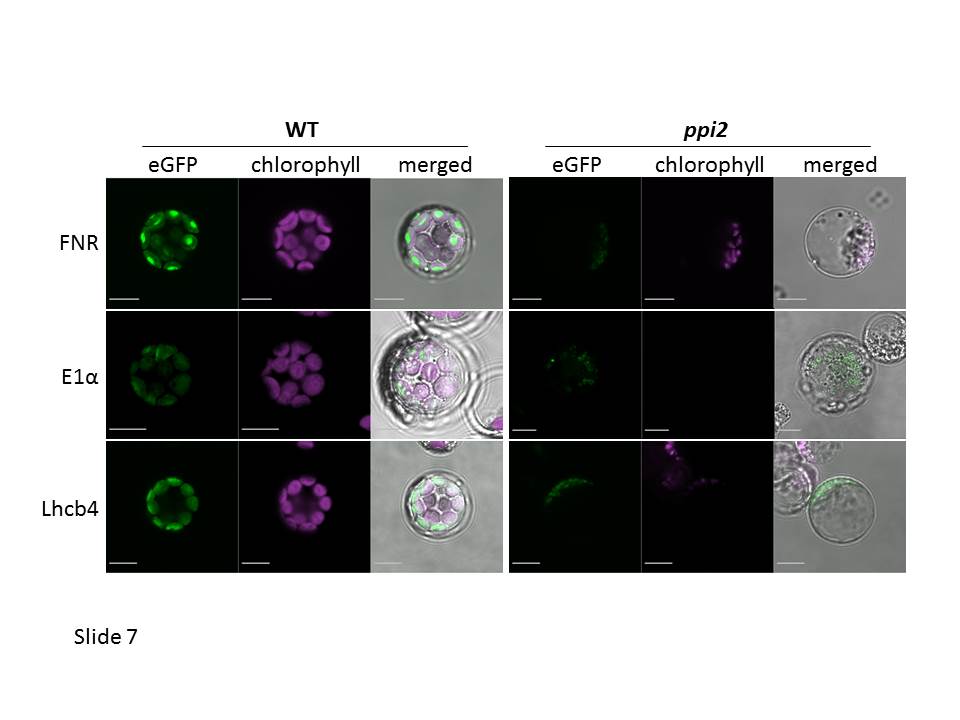


**Supplemental Figure S2: *in vivo* import of eGFP fusion proteins in the TOC159 deficient mutant *ppi2* and in wild type**

Microscopy of wild type and *ppi2* protoplasts, that were transiently transformed with FNR_1‑55_:eGFP, E1α_1‑100_:eGFP or Lhcb4_1‑100_:eGFP, bars = 10 µm.


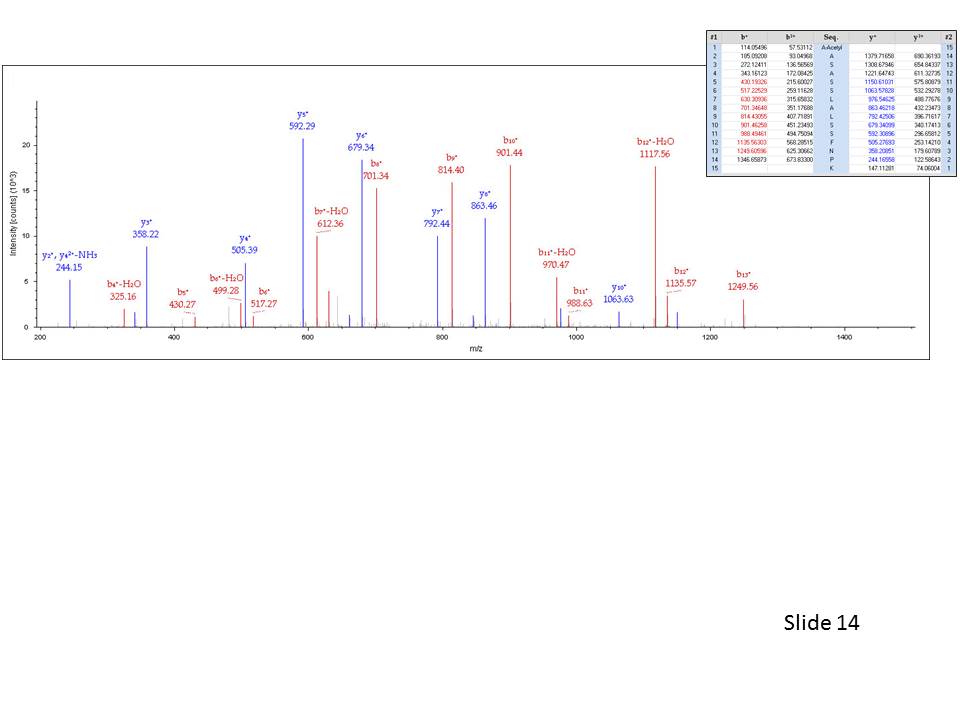

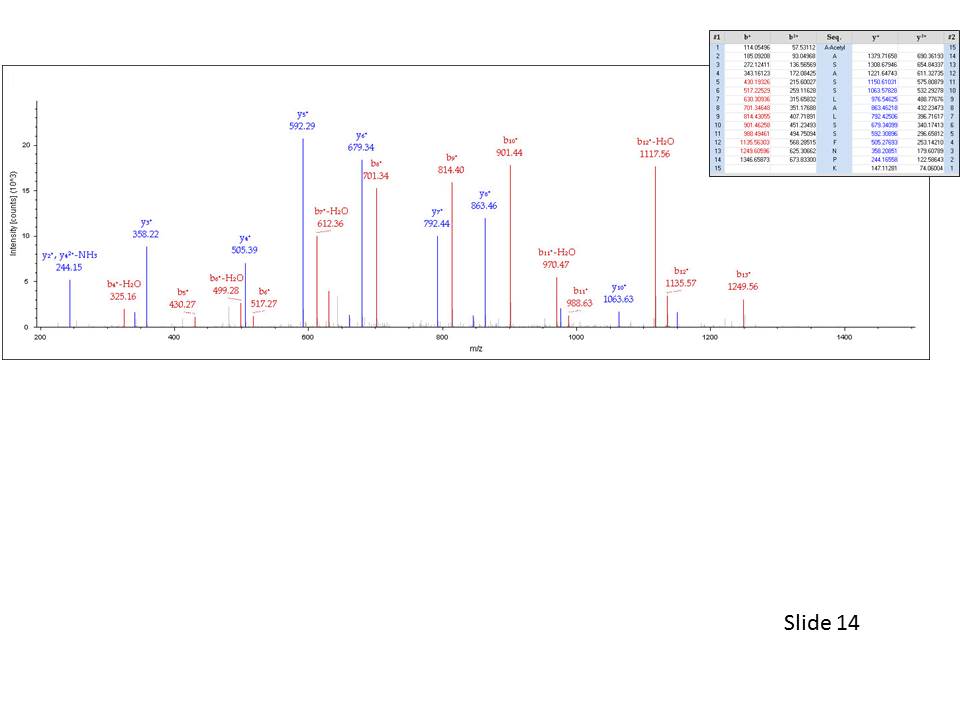


**A**

**B**

**C**


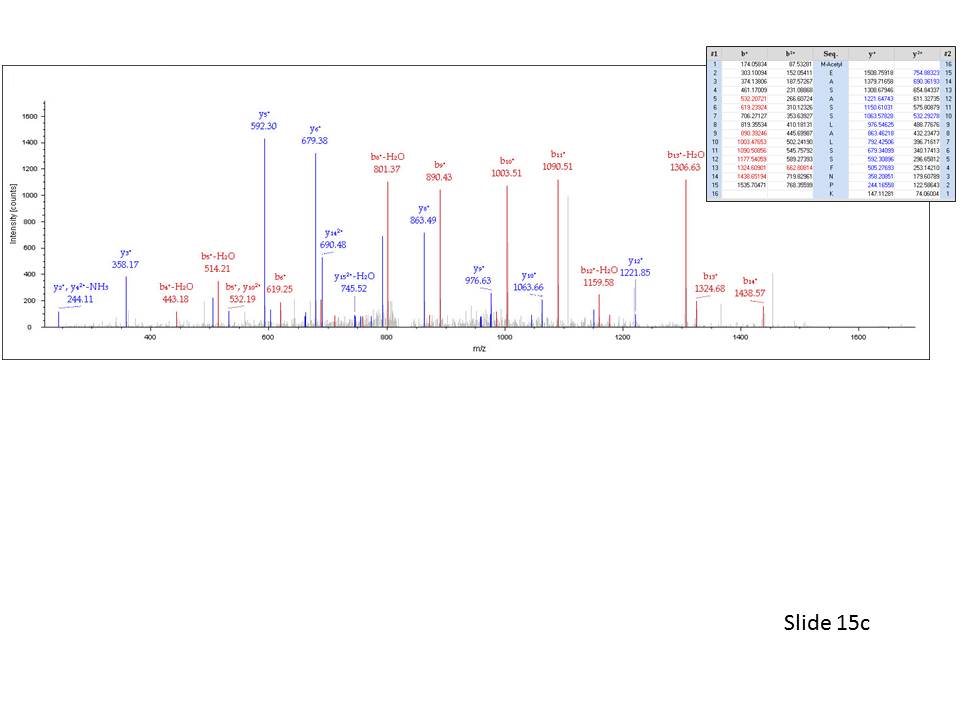

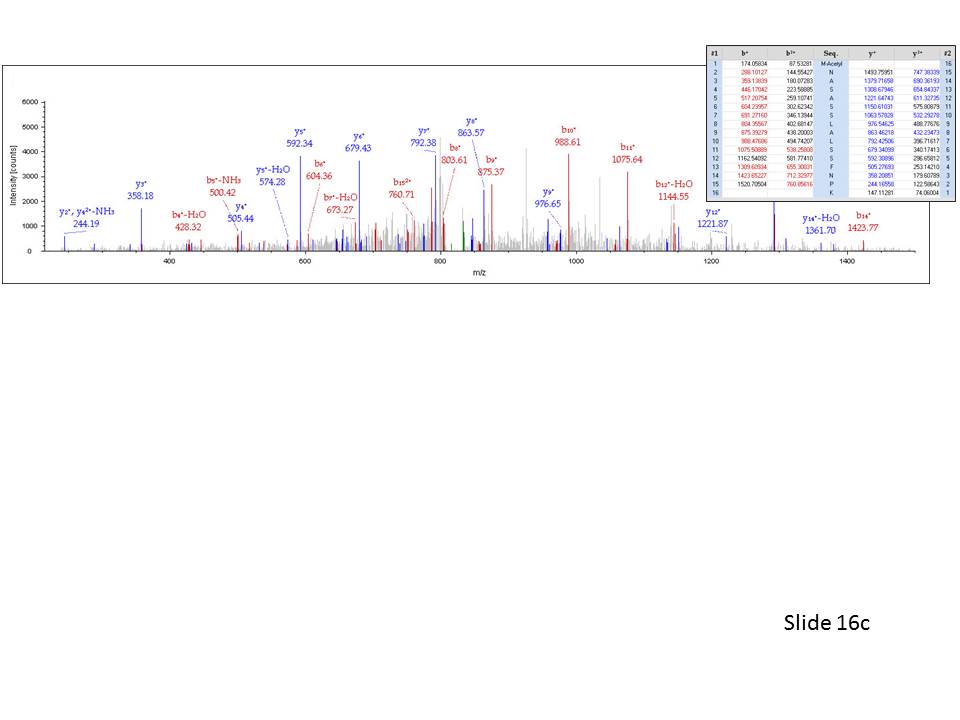


**Supplemental Figure S3: Annotated MS/MS spectra of the identified N-terminal peptides from RNP29**

MS/MS spectra of the N-terminal peptides of the authentic RNP29 (A), RNP29A2E construct (B) and RNP29A2N construct (C) including the tables with the annotated b- and y-ion series. The mass spectrometry data were obtained on an Orbitrap Velos.

**Supplemental Table S1: Nucleotide sequences of primers used for plasmid construction**

| RNP29_NcoI_for | CACCATGGCTGCTTCAGCTTCGTCT |
| --- | --- |
| RNP29A2E_NcoI_for | CACCATGGAGGCTTCAGCTTCGTCT |
| RNP29A2N_BspH1_for | CATCATGAACGCTTCAGCTTCGTCT |
| RNP29_NcoI_rev | CCATGGCGAAAGGAAGGTTACCAAC |
| Lhcb4_NcoI_for | CACCATGGCCGCAACATCC |
| Lhcb4_NcoI_rev | CCATGGCATTCTGGTCTAGTG |
| E1alpha_NcoI_for | CACCATGGCGACGGCTTTCGCTCCC |
| E1alpha_NcoI_rev | CCATGGCCATGTCTTCGAAAGATCTACC |
| Fd2_NcoI_for | CACCATGGCTTCCACTGCTCTCTCA |
| Fd2_NcoI_rev | CCATGGCAGCACAGCTCGAACAAGA |
